# Twelve New Genomic Loci Associated With Bone Mineral Density

**DOI:** 10.3389/fendo.2020.00243

**Published:** 2020-04-22

**Authors:** Lu Liu, Min Zhao, Zong-Gang Xie, Ju Liu, Hui-Ping Peng, Yu-Fang Pei, Hong-Peng Sun, Lei Zhang

**Affiliations:** ^1^Center for Genetic Epidemiology and Genomics, School of Public Health, Medical College of Soochow University, Jiangsu, China; ^2^Jiangsu Key Laboratory of Preventive and Translational Medicine for Geriatric Diseases, Soochow University, Jiangsu, China; ^3^Kunshan Hospital of Traditional Chinese Medicine, Jiangsu, China; ^4^The Second Affiliated Hospital of Soochow University, Jiangsu, China; ^5^Department of Epidemiology and Health Statistics, School of Public Health, Medical College of Soochow University, Jiangsu, China; ^6^Department of Children Health and Social Medicine, School of Public Health, Medical College of Soochow University, Jiangsu, China

**Keywords:** bone mineral density, genome-wide association study, osteoporosis, joint analysis, MTAG

## Abstract

Aiming to identify more genomic loci associated with bone mineral density (BMD), we conducted a joint association analysis of 2 genome-wide association study (GWAS) by the integrative association method multi-trait analysis of GWAS (MTAG). The first one is the single GWAS of estimated heel BMD (eBMD) in the UK biobank (UKB) cohort (*N* = 426,824), and the second one is the GWAS meta-analysis of total body BMD (TB-BMD) in 66,628 participants from 30 studies. Approximate conditional association analysis was performed in the identified novel loci to identify secondary association signal. Statistical fine-mapping was conducted to prioritize plausible credible risk variants (CRVs). Candidate genes were prioritized based on the analyses of cis- expression quantitative trait locus (cis-eQTL) and cis-protein QTL (cis-pQTL) information as well as the functional category of the SNP. By integrating the information carried in over 490,000 participants, this largest joint analysis of BMD GWAS identified 12 novel genomic loci at the genome-wide significance level (GWS, *p* = 5.0 × 10^−8^), nine of which were for eBMD and four were for TB-BMD, explaining an additional 0.11 and 0.23% heritability for the two traits, respectively. These loci include 1p33 (lead SNP rs10493130, p_eBMD_ = 3.19 × 10^−8^), 5q13.2 (rs4703589, p_eBMD_ = 4.78 × 10^−8^), 5q31.3 (rs9324887, p_TB−BMD_ = 1.36 × 10^−9^), 6p21.32 (rs6905837, p_eBMD_ = 3.32 × 10^−8^), 6q14.1 (rs10806234, p_eBMD_ = 2.63 × 10^−8^), 7q21.11 (rs10806234, p_TB−BMD_ = 3.37 × 10^−8^), 8q24.12 (rs11995866, p_eBMD_ = 6.72 × 10^−9^), 12p13.31 (rs1639122, p_eBMD_ = 4.43 × 10^−8^), 12p12.1 (rs58489179, p_eBMD_ = 4.74 × 10^−8^), 12q24.23 (rs75499226, p_eBMD_ = 1.44 × 10^−8^), 19q13.31 (rs7255083, p_TB−BMD_ = 2.18 × 10^−8^) and 22q11.23 (rs13056137, p_TB−BMD_ = 2.54 × 10^−8^). All lead SNPs in these 12 loci are nominally significant in both original studies as well as consistent in effect direction between them, providing solid evidence of replication. Approximate conditional analysis identified one secondary signal in 5q13.2 (rs11738874, p_conditional_ = 5.06 × 10^−9^). Statistical fine-mapping analysis prioritized 269 CRVs. A total of 65 candidate genes were prioritized, including those with known biological function to bone development (such as *FGF1, COL11A2* and *DEPTOR*). Our findings provide novel insights into a better understanding of the genetic mechanism underlying bone development as well as candidate genes for future functional investigation.

## Introduction

Osteoporosis is a common aging-related disease characterized by low bone mass and micro-architectural deterioration of bone tissue with a consequent increase in bone fragility and susceptibility to fracture (1). These later complications are associated with significant individual morbidity and related healthcare costs. Fifteen per cent of white people over 50 years old suffer osteoporotic fracture in their remaining lifetime, and the projected costs expended on this disease will exceed $25 billion in the United States alone by year 2025 (2). Therefore, a better understanding of the mechanisms underlying osteoporosis may help to develop medications for osteoporosis prevention and treatment.

The diagnosis of osteoporosis is made from the measurement of bone mineral density (BMD), which is a highly heritable trait with heritability ranging from 50 to 80% (3). Previous genome-wide associations studies (GWAS) and their meta-analyses have identified over 500 genomic loci for BMD, accounting for up to 20% phenotypic variation (4–17). Nonetheless, compared with the total 36% of GWAS-attributable heritability (14), there is still a large portion of “missing” heritability to be discovered by enlarged GWAS or more efficient analysis.

In the present study, aiming to identify more genomic loci that are responsible for BMD variation, we conduct a joint analysis of 2 GWAS analyses. The first one is the largest single GWAS study of estimated heel BMD (eBMD) in 426,824 UK biobank (UKB) participants (17), and the second one is the GWAS meta-analysis of total body BMD (TB-BMD) in 66,628 participants (15), which is so far the second largest study for BMD. This analysis integrates the information from an expanded size of over 490,000 participants, and therefore has the potential maximal statistical power of gene mapping to date. We further prioritize plausible functional variants and candidate genes for future experimental validation.

## Materials and Methods

We performed a joint analysis of summary statistics from two large-scale BMD GWAS studies. No new ethnic approval was required.

### Study Samples

Two studies were incorporated into this joint association analysis. The first one is the single GWAS of eBMD in the UKB cohort (*N* = 426,824). In brief, the UKB sample is a large prospective cohort study of ~500,000 participants from across the United Kingdom, aged between 40 and 69 at recruitment. Heel BMD was estimated based on quantitative ultrasound speed of sound (SOS) and broadband ultrasound attenuation (BUA). Genome-wide genotypes were available for all participants at 784,256 genotyped autosome markers, and were imputed into UK10K haplotype, 1000 Genomes project phase 3 and Haplotype Reference Consortium (HRC) reference panels by IMPUTE2 (18). After quality controls, 426,824 qualified participants were used in GWAS analysis (17).

The second one is the GWAS meta-analysis of TB-BMD in 66,628 participants from 30 studies (15). In brief, TB-BMD was measured by dual energy X-ray absorptiometry (DXA). All participants had genome-wide genotype data and were imputed into the 1,000 Genomes project phase 1 or the combined 1,000 Genomes project phase 3 and the UK10K reference panel. Almost all popular imputation methods were used across studies (Supplementary Table 1). In their analyses, association was performed in each individual study, and the summary statistics across the 30 studies were meta-analyzed by a fixed-effects model.

A total of 1,553 participants from the UKB cohort were included in the TB-BMD study, accounting for 2.3 and 0.4% of TB-BMD and eBMD sample sizes, respectively. Association summary statistics for both studies were publicly available at the genetic factors for osteoporosis consortium (GEFOS) website (http://www.gefos.org), and were downloaded for analysis.

### SNP Inclusion Criteria

The SNP inclusion criteria were same as described previously (19). In brief, in each study, non-SNP variants and ambiguous SNPs (i.e., multiple SNPs corresponding to one single identifier) were excluded. In addition, SNPs presenting significant meta-analysis genetic heterogeneity (I^2^ > 50% or Q p-value < 0.1) in the TB-BMD study were excluded. After quality control (QC), 8,680,009 SNPs are available in both studies. After removing SNPs that are not concordant between the two studies (i.e., A/G vs. T/G polymorphisms), a total of 7,369,691 SNPs in common to both studies were used in subsequent analyses.

### Joint Association Analysis

The recently developed multi-trait analysis of GWAS (MTAG) method was used for joint association analysis, which accounts for sample overlap and trait heterogeneity between studies (20). In brief, MTAG estimates per SNP effect size for each trait by incorporating information contained in other correlated traits, and therefore has potential to improve statistical power of association test. MTAG takes summary statistics from multiple studies as input. The output of MTAG contains re-estimated effect size and *p*-value for each SNP in each trait.

The linkage disequilibrium score regression (LDSC) method was applied to the MTAG results to estimate the amount of genomic inflation due to confounding factors such as population stratification and cryptic relatedness (21). LDSC takes GWAS summary statistics as input and partitions overall inflated association statistic into one part attributable to polygenic architecture and another part due to population stratification and cryptic relatedness. Reference LD scores for the European population were downloaded from the software website (https://github.com/bulik/ldsc). The relative contribution of confounding factors was measured by attenuation ratio (AR), which is defined as (intercept-1)/(mean chi^2^ − 1), where intercept and mean chi^2^ are estimates of confounding and the overall association inflation, respectively (21).

Genome-wide significance (GWS) level was set to be 5.0 × 10^−8^. An independent locus was defined as one 1-MB region, which consists of two 500 kb regions from the lead SNP to both directions. A novel locus was declared if it was neither reported in previous GWAS studies nor significant at the GWS level in eBMD or TB-BMD study.

Individual variant effect size was estimated with the formula 2*f* (1–*f*)β^2^, where *f* is allele frequency and β is regression coefficient, which was estimated by MTAG.

### Approximate Conditional Analysis

To identify additional signals in regions of association, approximate conditional association analysis was performed in each region using the genome-wide complex trait analysis (GCTA) tool (22). A reference sample of 100,000 unrelated participants from the UKB cohort was generated for estimating LD pattern. Specifically, a total of 369,968 unrelated participants were inferred with kinship-based inference for GWAS (KING) software (23), from whom the 100,000 participants of the reference sample were randomly drawn.

A recursive conditional association analysis was performed. In each iteration, an approximate conditional analysis conditioning on the current list of lead variants was performed. A secondary significant variant was defined at the conditional GWS level (conditional *p* < 5 × 10^−8^). The variant with the smallest *p*-value among such identified ones was added into the list of lead variants. Iterations of the conditional analysis were run until no significant signal can be identified.

### Statistical Fine-Mapping of Credible Risk Variants

Statistical fine-mapping of credible risk variants (CRVs) was performed with FINEMAP (24). FINEMAP uses GWAS summary statistics and applies a shotgun stochastic search algorithm to efficiently exploring a set of most important causal configurations in the associated region. It relies on a matched reference panel for LD estimation. Again, the above reference panel of 100,000 unrelated UKB participants was used for LD estimation with LDstore (25). Software parameters were set by default. The outputs of FINEMAP include the posterior probability of each SNP being causal. For each locus, we sorted the posterior probabilities in an descending order, and constructed the set of CRVs by including those SNPs whose posterior probabilities were within one order of magnitude of the largest posterior probability.

### Candidate Gene Prioritization

We prioritized candidate genes in the identified novel loci by annotating the CRVs for their cis-expression quantitative trait locus (cis-eQTL) and cis-protein QTL (cis-pQTL) effects, and for their distances to genes.

Cis-eQTL effect was assessed from two datasets. The first one is the 44 tissues from the GTEx project (v6) (26). Pre-compiled cis-eQTL results were downloaded from the GTEx web portal (www.gtexportal.org/). The distance between the SNP and the transcription starting site (TSS) of the target gene was assumed to be <500 kb. Assuming a maximal number of 5,000 independent variants over such a 1-MB region, significant cis-eQTL was declared at *p* < 1.0 × 10^−5^ (0.05/5,000). The second one is the lymphoblastoid cell lines of 373 European individuals from the 1,000 genomes project (27). Pre-compiled cis-eQTL results were downloaded from the study website (https://www.ebi.ac.uk/Tools/geuvadis-das/). Significant cis-eQTL was declared under the same criteria.

Cis-pQTL information was accessed from Sun et al. (28). In that largest proteome study to date, the authors measured plasma protein levels of 3,301 healthy individuals using the SOMAscan platform (SomaLogic, Inc., Boulder, Colorado, USA) comprising 4,034 distinct aptamers (SOMAmers) covering 3,623 proteins. GWAS summary statistics for 3,284 proteins were downloaded from the study's website. Cis-pQTL was searched within 500 kb distance from a target gene. Analogously to the cis-eQTL analysis, significant cis-pQTL was declared at *p* < 1.0 × 10^−5^.

SNPs were annotated by variant effect predictor (VEP) for their functional category (29). SNP-gene distance was annotated by prioritization of candidate causal genes at molecular QTLs (ProGeM) software (30). Genes nearest, second nearest and third nearest to each lead SNP were listed.

## Results

### Main Association Results

There are 13,753,401 and 18,259,434 SNPs in the eBMD and TB-BMD studies. After QC, 7,369,691 SNPs present in both studies were qualified for analysis. Genetic correlation coefficient was reported to be 0.57 (19), implying shared BMD heritability between the two studies. In the MTAG analysis of the eBMD study, the intercept and mean chi-square are 1.09 and 3.30, respectively, suggesting that 96.1% of the inflation in the mean chi-squared statistic is from polygenic architecture rather than from population stratification. Similarly, the intercept and mean chi-square in the MTAG analysis of the TB-BMD study are 0.78 and 1.84, respectively, suggesting that most of the inflation is from polygenic architecture.

The original UKB eBMD study summary statistics contain 76,400 SNPs significant at the GWS level, encompassing 659 distinct loci. In the MTAG analysis, all the 659 lead SNPs are consistent in effect direction with original ones. Among them, 556 (84.4%) remain significant at the GWS level. Though the other 103 lead SNPs become non-significant at the GWS level, they are all nominally significant (*p* < 0.05) with *p*-value ranging from 5.02 × 10^−8^ to 8.88 × 10^−5^. Of the 556 GWS significant SNPs, *p*-value at 193 (34.7%) SNPs gets smaller while those at 363 SNPs gets higher.

The original GEFOS TB-BMD study summary statistics contain 3,842 SNPs significant at the GWS level, encompassing 68 distinct loci. In the MTAG analysis, all the 68 lead SNPs are consistent in effect direction with original ones. Among them, 59 (86.8%) remain significant at the GWS level, while the other nine become non-significant at the GWS level. Only one SNP rs1037011 becomes non-significant even at the loosely nominal significance level (*p* = 0.07). Interestingly, this SNP is GWS significant in both original studies (*p*_eBMD_ = 3.40 × 10^−9^, *p*_TB−BMD_ = 1.54 × 10^−12^), but the effect allele T has opposite direction (β_eBMD_ = 0.01, β_TB−BMD_ = −0.04). The possible reason for the opposite directions could be strand alignment error or true opposite genetic effects, pending further investigation. Of the 59 GWS significant SNPs, *p*-value at up to 53 SNPs gets smaller while that at the remaining 6 SNPs gets higher.

**Figure 1:**
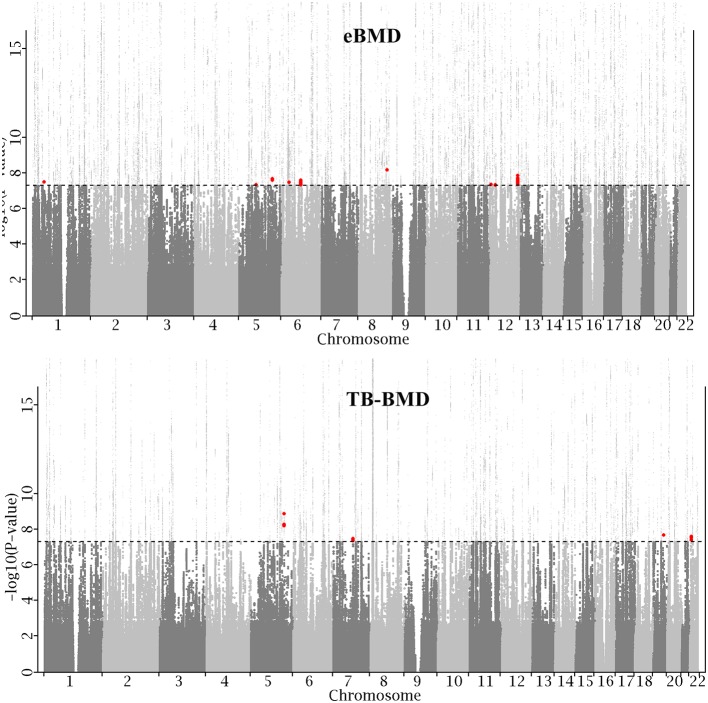
Manhattan plot. X-axis represents genomic position on chromosome and Y-axis represents −log10 (*P*-value). For ease of presentation, Y-axis is truncated at 15. Dotted line represents genome-wide significance level (GWS, 5.0 × 10^−8^). All known loci, including GWS loci in either eBMD (top) or TB-BMD (bottom) study, and previously reported loci that were retrieved from the EBI GWAS catalog, are plotted in light gray. Newly identified loci are plotted in red.

To search for additional loci, we evaluated MTAG results of all SNPs excluding those within 500 kb of a locus that was either GWS significant in either original study or was reported previously. This identified 225 SNPs in the eBMD study and 15 SNPs in the TB-BMD study. To increase the confidence of association results, only SNPs of *p*-value < 0.05 in both original studies were kept, resulting in 79 SNPs in the eBMD study and again 15 SNPs in the TB-BMD, encompassing nine and four distinct loci, respectively. Only one locus overlapped between the two studies, resulting in a total number of 12 distinct novel loci. All the 12 lead SNPs are consistent in effect direction in both original studies. Manhattan plot is displayed in Figure 1 and main results are listed in Table 1. Regional plots at all the novel loci are displayed in Supplementary Figure 1. The 12 lead SNPs collectively explain 0.11 and 0.23% phenotypic variance for eBMD and TB-BMD, respectively.

**Table 1:** Main results of the identified novel loci.

					**eBMD (*****N*** **= 426,824)**						**TB-BMD**						
					**Original**			**MTAG**			**Original**				**MTAG**		
**SNP**	**CHR**	**BP**	**EA/OA**	**EAF**	**BETA**	**SE**	***P***	**BETA**	**SE**	***P***	**BETA**	**SE**	***P***	***N***	**BETA**	**SE**	***P***
rs10493130	1	48,341,005	G/A	0.68	−0.01	0.002	4.20E-7	−0.01	0.003	**3.19E-8**	−0.02	0.006	5.85E-3	66,546	−0.02	0.004	4.97E-6
rs4703589	5	72,097,351	T/C	0.47	0.01	0.002	2.20E-6	0.01	0.002	**4.78E-8**	0.02	0.006	9.67E-4	66,604	0.02	0.004	1.32E-6
rs9324887	5	142,047,831	A/G	0.72	−0.01	0.002	2.00E-5	−0.02	0.003	**2.20E-8**	−0.03	0.006	1.57E-7	66,480	−0.03	0.004	**1.36E-9**
rs6905837	6	32,626,205	C/T	0.95	−0.03	0.004	2.00E-7	−0.03	0.006	**3.32E-8**	−0.05	0.019	4.21E-3	37,410	−0.05	0.010	2.89E-6
rs10806234	6	82,685,299	A/G	0.40	−0.01	0.002	4.70E-6	−0.01	0.002	**2.63E-8**	−0.02	0.006	3.78E-5	66,048	−0.02	0.004	6.51E-8
rs1019203	7	84,784,053	C/T	0.64	0.01	0.002	3.60E-5	0.01	0.003	8.58E-8	0.03	0.006	5.21E-6	66,553	0.02	0.004	**3.37E-8**
rs11995866	8	121,058,098	G/A	0.61	−0.01	0.002	6.80E-7	−0.01	0.002	**6.72E-9**	−0.01	0.006	0.04	66,569	−0.02	0.004	1.94E-5
rs1639122	12	6,711,147	C/A	0.56	−0.01	0.002	6.30E-7	−0.01	0.002	**4.43E-8**	−0.02	0.006	7.85E-3	66,038	−0.02	0.004	7.27E-6
rs58489179	12	25,525,053	G/A	0.91	0.02	0.003	1.10E-7	0.02	0.004	**4.74E-8**	0.04	0.012	2.32E-3	63,629	0.03	0.007	2.59E-6
rs75499226	12	120,515,773	C/A	0.91	0.02	0.003	7.60E-7	0.02	0.004	**1.44E-8**	0.03	0.009	4.85E-3	66,572	0.03	0.006	2.96E-6
rs7255083	19	44,337,803	T/C	0.57	−0.01	0.002	7.30E-6	−0.01	0.002	1.77E-7	−0.03	0.006	2.08E-6	66,595	−0.02	0.004	**2.18E-8**
rs13056137	22	23,407,261	C/A	0.74	0.01	0.002	4.60E-6	0.01	0.003	7.01E-8	0.03	0.006	5.23E-6	66,419	0.02	0.004	**2.54E-8**

*CHR, chromosome; BP, base-pair, based on GRCH37 genome assembly; EA, effect allele; OA, other allele; EAF, effect allele frequency that was derived from the UKB study summary results; BETA, regression coefficient; SE, standard error of BETA; N, sample size; P, p-value. P-values less than the genome-wide significance (GWS, 5.0 × 10^-8^) were marked in bold*.

In Supplementary Table 2, we listed genomic distance and LD value (*r*^2^) between the lead SNP at each of the identified novel loci and the nearest GWS SNP at the nearest known locus. The results show that all pairs of SNPs are in complete linkage equilibrium, confirming the novelty of the identified loci.

### Approximate Conditional Analysis

Using GCTA, we performed an approximate conditional analysis. In the eBMD analysis, one secondary signal was identified at rs11738874 (p_MTAG_ = 5.38 × 10^−8^, p_conditional_ = 5.06 × 10^−9^). This SNP is significant in both original studies (p_eBMD_ = 7.60 × 10^−6^, p_TB−BMD_ = 7.30 × 10^−3^). It is in linkage equilibrium with the lead SNP rs4703589 (LD *r*^2^ = 0.0), indicating an independent association signal.

No secondary associations were found in the TB-BMD analysis.

### Credible Risk Variants

With FINEMAP, we performed a statistical fine-mapping analysis. In the eBMD analysis, 214 CRVs were prioritized at the 12 novel loci, with an average of 18 variants per locus. The locus with most number of variants is 12q24.23, in which up to 38 variants were prioritized. There is one locus at 19q13.31 with only one causal variant being prioritized, which is the lead SNP rs7255083. Its posterior probability is up to 0.87, being 15-fold larger than that of the second variant rs2356401 (posterior probability 0.06) (Supplementary Table 3).

In the TB-BMD analysis, 64 variants are prioritized at 12 loci, with an average of 5 variants per locus. The locus with most number of variants is again 12q24.23, in which 7 variants are prioritized. No locus is found to have only one causal variant (Supplementary Table 3).

The two sets of CRVs derived from both analyses were merged into one single set of 269 CRVs, where 9 CRVs overlap between the two analyses.

### Newly Identified Loci/Genes

We next prioritized candidate genes based on the annotations of cis-eQTL and cis-pQTL effects and functional categories in the above set of CRVs.

In the 44 tissues of the GTEx (v6) dataset, 116 CRVs from 10 regions are cis-associated with the expression of nearby genes at a variety of tissues. The two regions with no evidence of cis-eQTL effect are 6q14.1 and 7q21.11 (Supplementary Table 4).

In the lymphoblastoid cell lines, 12 variants from four regions are associated with nearby gene expressions, where seven variants overlapped with the GTEx variants. There are three associated genes that are not found in the list of GTEx prioritized genes (Supplementary Table 4).

In the pQTL dataset, multiple SNPs at 12p13.31 are cis- associated with *TAPBPL* (TAP Binding Protein Like) protein level. The most significant association is observed at rs1639122 (*p* = 3.31 × 10^−43^), which is a mis-sense mutation in a nearby gene *CHD4* (Chromodomain Helicase DNA Binding Protein 4). Another SNP rs11609726 at this region is also associated with one additional protein *C1RL* (Complement C1r Subcomponent Like, *p* = 4.36 × 10^−8^) (Supplementary Table 5).

Combining the various annotations, a total of 65 candidate genes were prioritized at the 12 novel loci (Table 2).

**Table 2:** Prioritized candidate genes at the identified novel loci.

**Locus**	**Lead SNP**	**CHR**	**BP**	**Gene**
1p33	rs10493130	1	48,341,005	SLC5A9(E), SKINTL(E), SPATA6(E), TRABD2B(N)
5q13.2	rs4703589	5	72,097,351	MAP1B(E), FCHO2(E,S), TNPO1(N), ZNF366(T)
5q31.3	rs9324887	5	142,047,831	FGF1(E,N), ARHGAP26(S), SPRY4(T)
6p21.32	rs6905837	6	32,626,205	CYP21A1P(E), HCG23(E), TNXA(E), PRRT1(E), PSMB9(E), TAP1(E), COL11A2(E), RPL32P1(E), HLA-DQB1(N), HLA-DQA1(S), HLA-DRB1(T)
6q14.1	rs10806234	6	82,685,299	IBTK(N), FAM46A(S), TPBG(T)
7q21.11	rs1019203	7	84,784,053	SEMA3D(N)
8q24.12	rs11995866	8	121,058,098	DSCC1(E), DEPTOR(E,N), COL14A1(S), DSCC1(T)
12p13.31	rs1639122	12	6,711,147	TAPBPL(E,P), MRPL51(E), LRRC23(E), CHD4(E,N), C1RL(P), LPAR5(S), NOP2(T)
12p12.1	rs58489179	12	25,525,053	LRMP(E), CASC1(E), IFLTD1(N), KRAS(S)
12q24.23	rs75499226	12	120,515,773	PRKAB1(E), TMEM233(E), CCDC64(E,N), COQ5(E), POP5(E), GATC(E), RAB35(S), GCN1L1(T)
19q13.31	rs7255083	19	44,337,803	ZNF575(E), XRCC1(E), PHLDB3(E), LYPD3(E), PINLYP(E), ZNF283(E,N), ZNF155(E), ZNF223(E), ZNF404(E,T), ZNF45(E), LYPD5(S)
22q11.23	rs13056137	22	23,407,261	BCR(E), RAB36(E,T), GNAZ(E,S), FBXW4P1(E), RTDR1(N)

*Genes were prioritized as following: gene nearest to the lead SNP (N); gene second nearest to the lead SNP (S); gene third nearest to the lead SNP (T); gene with mRNA level in association with one or more CRVs (cis-eQTL, E); gene with protein level in association with one or more CRVs (cis-pQTL, P)*.

## Discussion

By conducting a joint association study of 2 large-scale GWAS analyses, the present study integrated the information contained in over 490,000 participants, making it the largest BMD GWAS analysis to date. As a result, 12 new genomic loci were identified, demonstrating the enhanced statistical power.

Because the two integrated traits are not same, naive meta-analysis is not applicable. Instead, we used the recently developed method MTAG for integration analysis. MTAG is applicable to analyzing genetically correlated traits. It uses two sources of information to integrate association signals. The first one is that the true effect of target SNP is correlated across traits, and the second one is that the estimation error of the SNP' effects is correlated across traits (20). When two GWAS summary statistics datasets have overlapped individuals, for example, shared control individuals, MTAG is indeed capable of handling this relatedness. It accomplishes this by applying the bivariate LD score regression to estimating the correlation in GWAS estimation error due to sample overlap. In our analysis, 1,533 participants overlapped between the eBMD and the TB-BMD studies, accounting for 0.36% of the total UKB participants. This tiny portion of overlap is not expected to have a major effect on the results, let alone that MTAG analysis took the overlap into account.

We were able to identify only one bone-related dataset, which is the lymphoblastoid cell lines. Only 12 CRVs from this dataset were found to exert cis-eQTL activity. The use of the 44 GTEx tissues was not motivated by their specific relationship to BMD, but was by a maximal coverage of available cis-eQTL SNPs. Because while some cis-eQTL activities are tissue-specific, some others are common across tissues. For example, Of the 12 cis-eQTL variants identified from the lymphoblastoid cell lines, seven overlapped with the GTEx cis-eQTL variants, implying that a considerable portion of cis-eQTL sites were common across tissues.

The prioritized candidate genes include those being linked to bone biology in previous literature. At 5q31.3, for example, the lead SNP rs9324887 is located in the intron of *FGF1* (Fibroblast Growth Factor-1) gene and is associated with its expression. *FGF1* is a member of the *FGF* signaling pathway that participates in the regulation of bone development (31). Local and systemic *FGF1* increases new bone formation and bone density. It also appears to restore bone micro-architecture and prevent bone loss associated with estrogen-withdrawal (32). At 6p21.32, *COL11A2* (Collagen Type XI Alpha 2 Chain) is one of the prioritized genes by cis-eQTL analysis. It is a member of the collagen family of extracellular proteins. It is a critical positive factor in the regulation of extra cellular matrix (ECM), which mineralizes to bone (33, 34). At 8q24.12, two genes *DSCC1* (DNA Replication And Sister Chromatid Cohesion 1) and *DEPTOR* (DEP Domain Containing MTOR Interacting Protein) were prioritized. It was only recently that *DEPTOR* was found to play a novel function in osteogenic differentiation by inhibiting *MEG3*-mediated activation of *BMP4* signaling, suggesting its involvement in osteoporosis (35).

In addition to those established candidate genes to bone biology, we also prioritized multiple candidate genes with no known function to bone metabolism. Among them, *SEMA3D* (Semaphorin 3D) at 7q21.11 belongs to a member of the semaphorin III family of secreted signaling proteins that are involved in axon guidance during neuronal development (36). Despite the lack of evidence for its involvement in bone development, one of its paralogs, *SEMA3A* (Semaphorin 3A), is found to regulate bone remodeling indirectly by modulating sensory nerve development instead of by acting on osteoblasts (37). In addition, *SEMA3A* and *SEMA3E* (Semaphorin 3E), are also reported to be associated with hypogonadotropic hypogonadism (38, 39). Hypogonadotropic hypogonadism is known to regulate bone density (40). These lines of evidence imply *SEMA3D* may have a regulatory role on bone development.

In conclusion, by conducting a joint analysis of two large-scale genome-wide association study and meta-analysis, we have identified 12 novel loci associated with BMD. Our findings provide candidate genes for future functional investigations and for a better understanding of the genetic mechanism underlying bone development.

## Data Availability Statement

Publicly available datasets were analyzed in this study. This data can be found here: http://www.gefos.org/?q=content/gefos-lifecourse-tb-bmd-gwas-results, http://www.gefos.org/?q=content/data-release-2018.

## Author Contributions

LZ was in charge of conceptualization and design. LL, MZ, and Z-GX contributed to data collection, formal analysis, and manuscript drafting. JL and H-PP contributed to the literature review and manuscript editing. Y-FP, H-PS, and LZ are responsible for supervision and funding. All authors approved the final version to be published.

## Conflict of Interest

The authors declare that the research was conducted in the absence of any commercial or financial relationships that could be construed as a potential conflict of interest.
