# Supplementary figures and images for: Twelve New Genomic Loci Associated With Bone Mineral Density
Source: Front Endocrinol (Lausanne). 2020 Apr 22;11:243. doi: 10.3389/fendo.2020.00243 (PMC7188784; doi:10.3389/fendo.2020.00243)

# Supplemental Figure 1. Regional plot

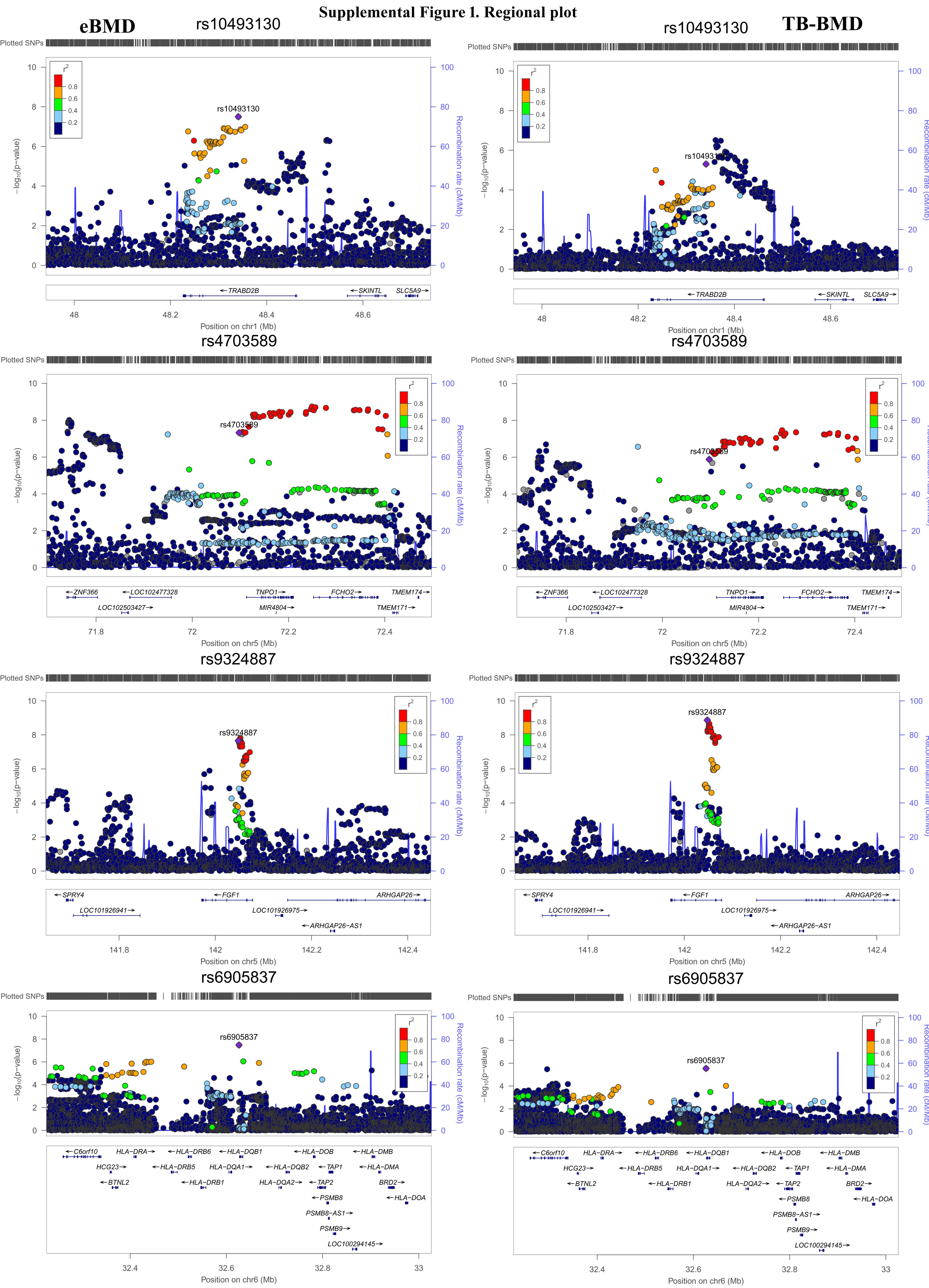

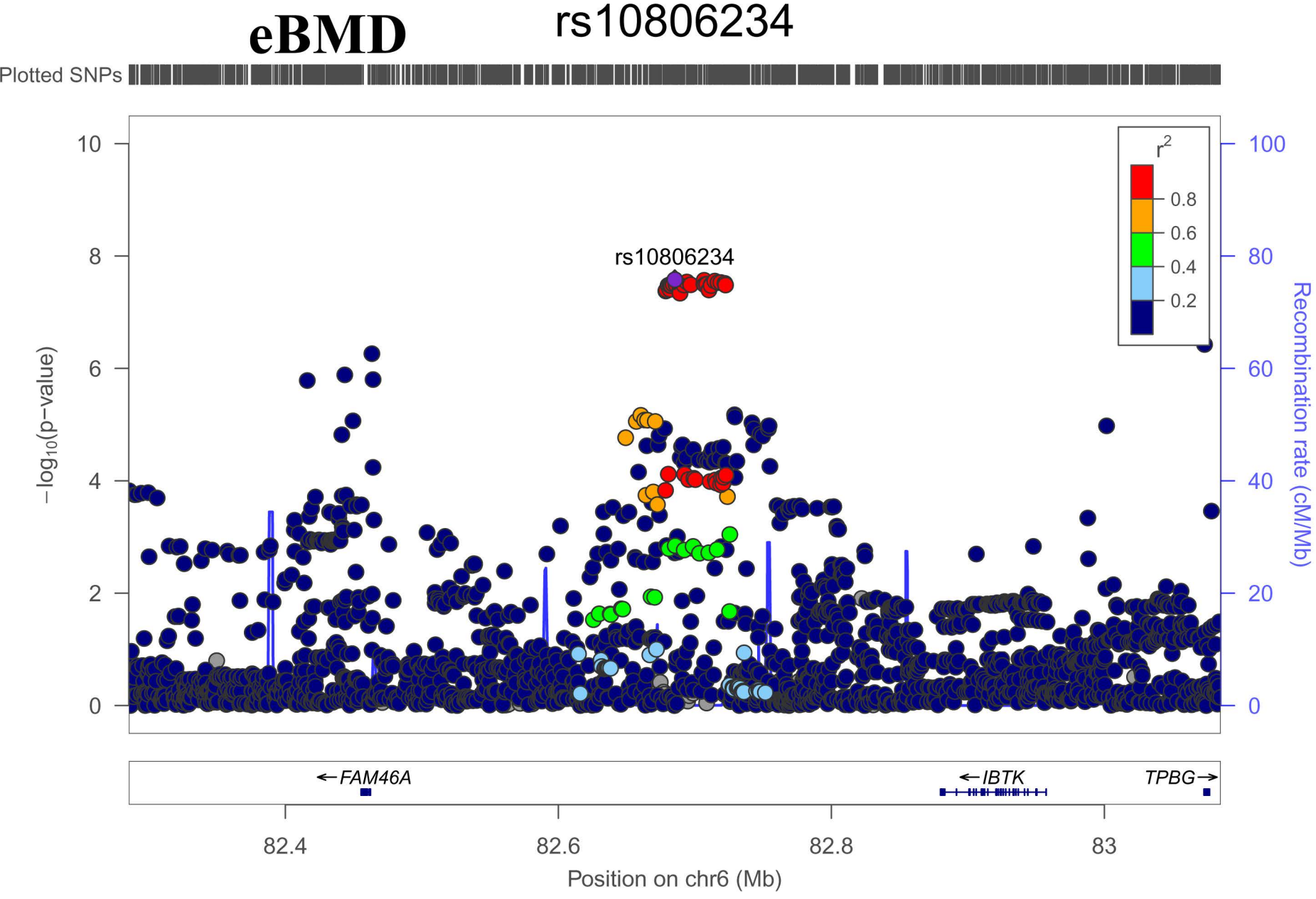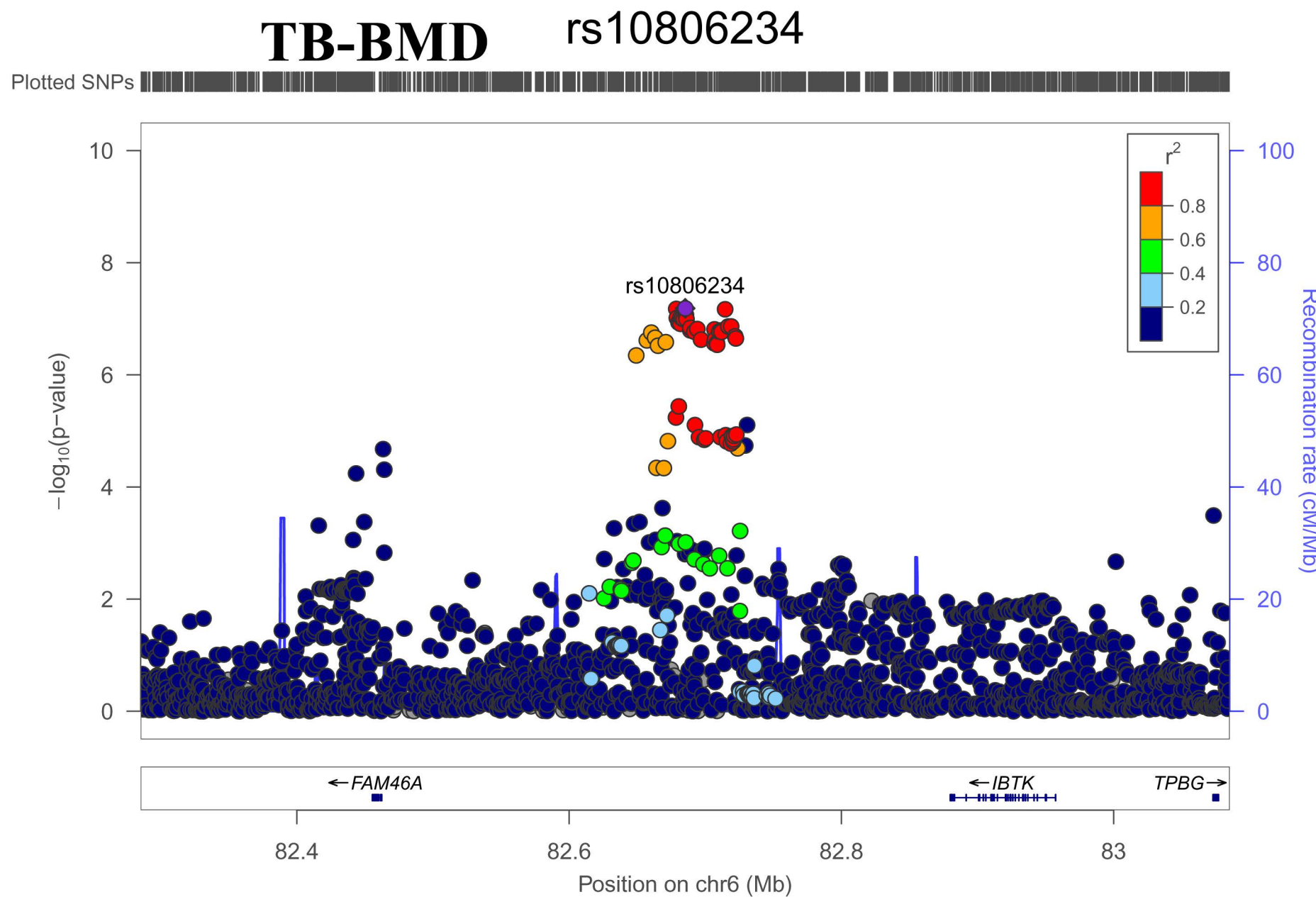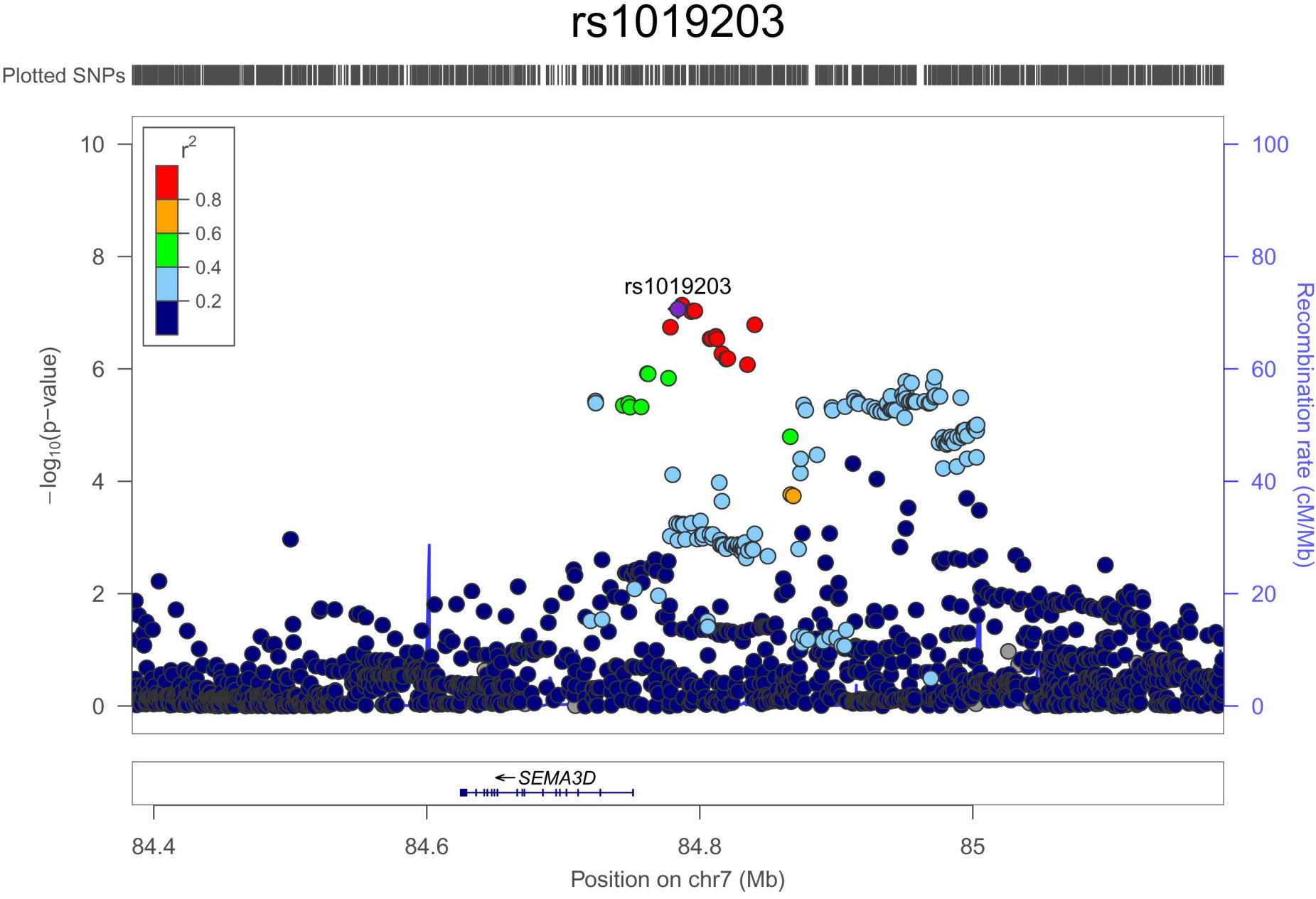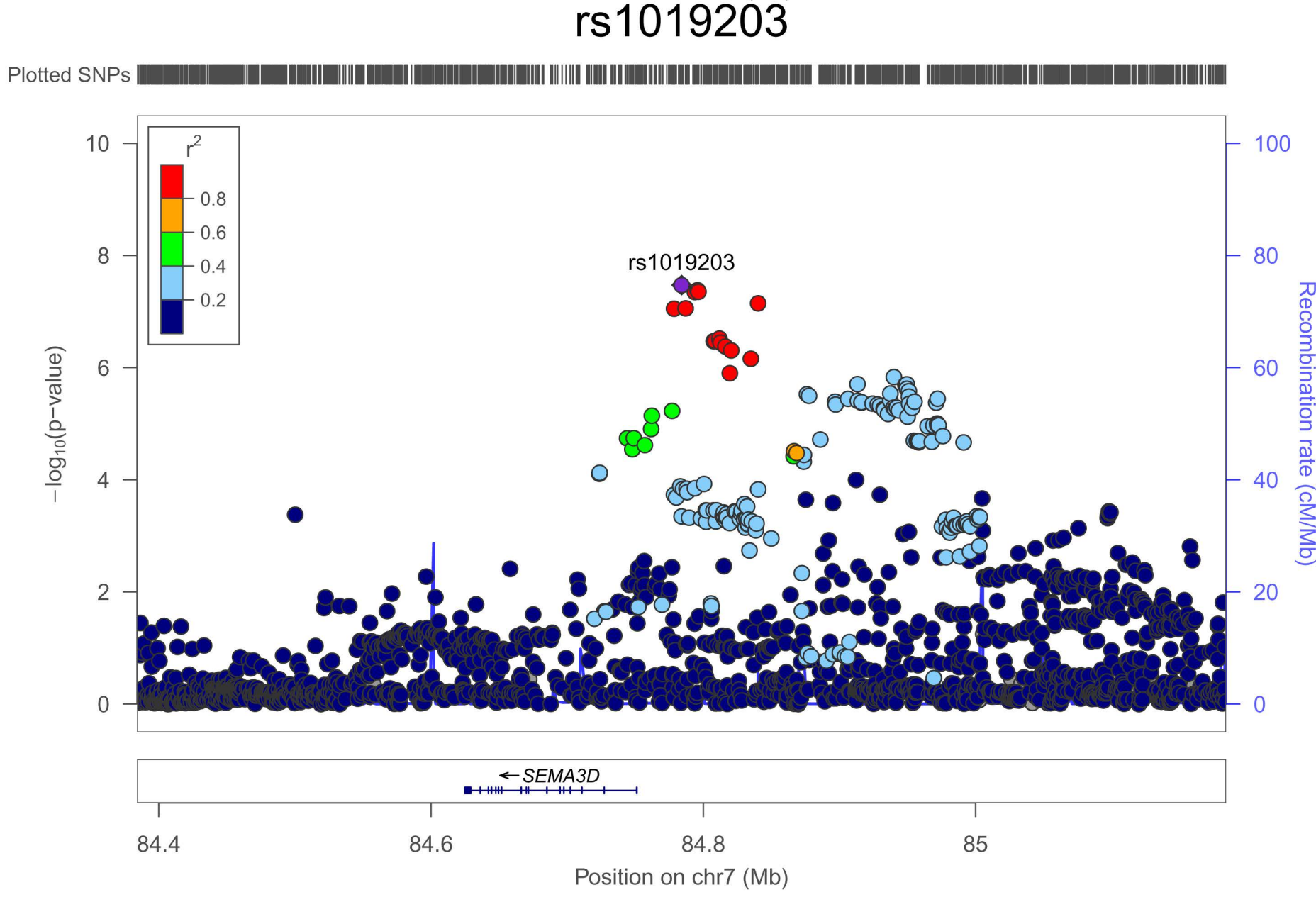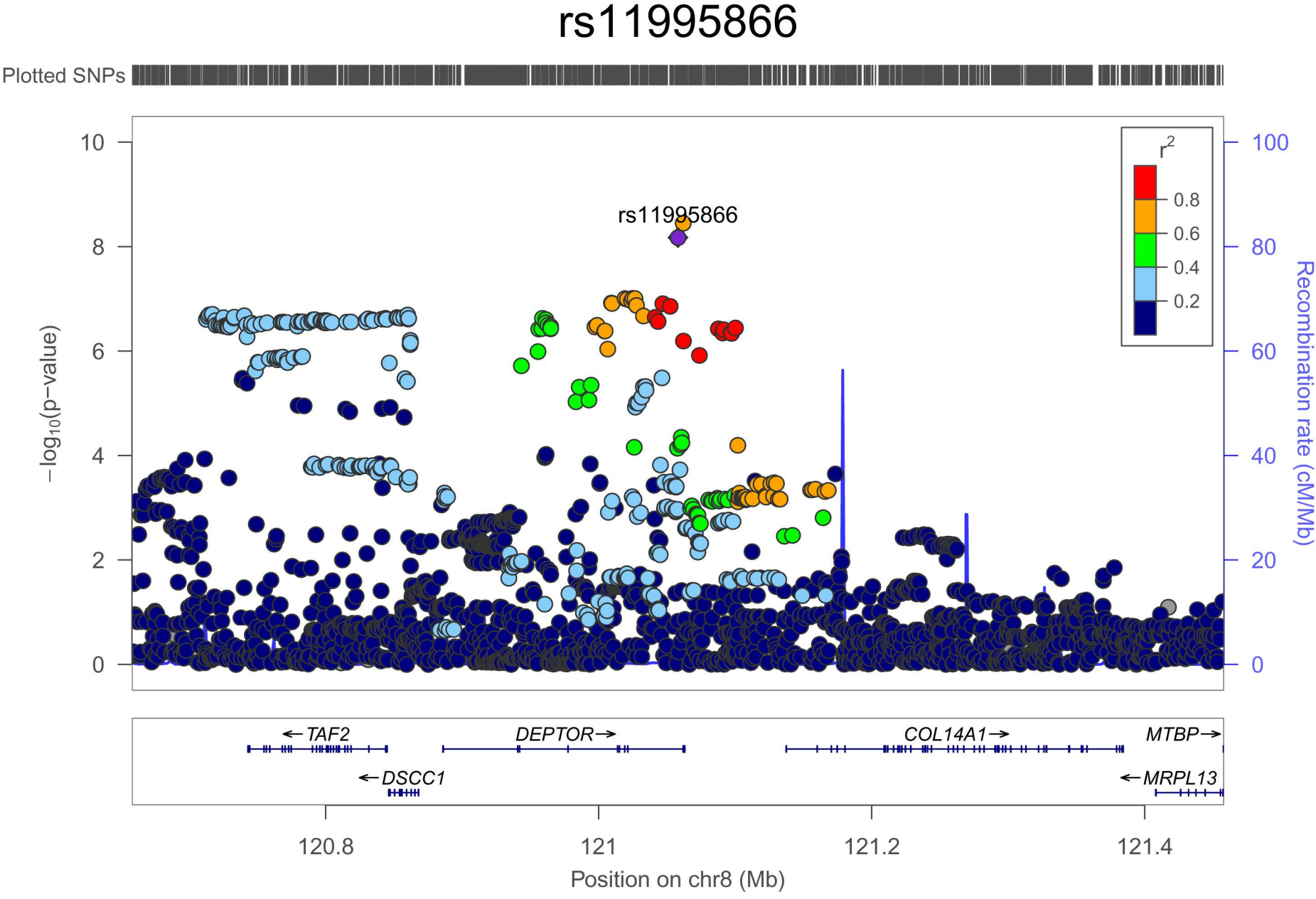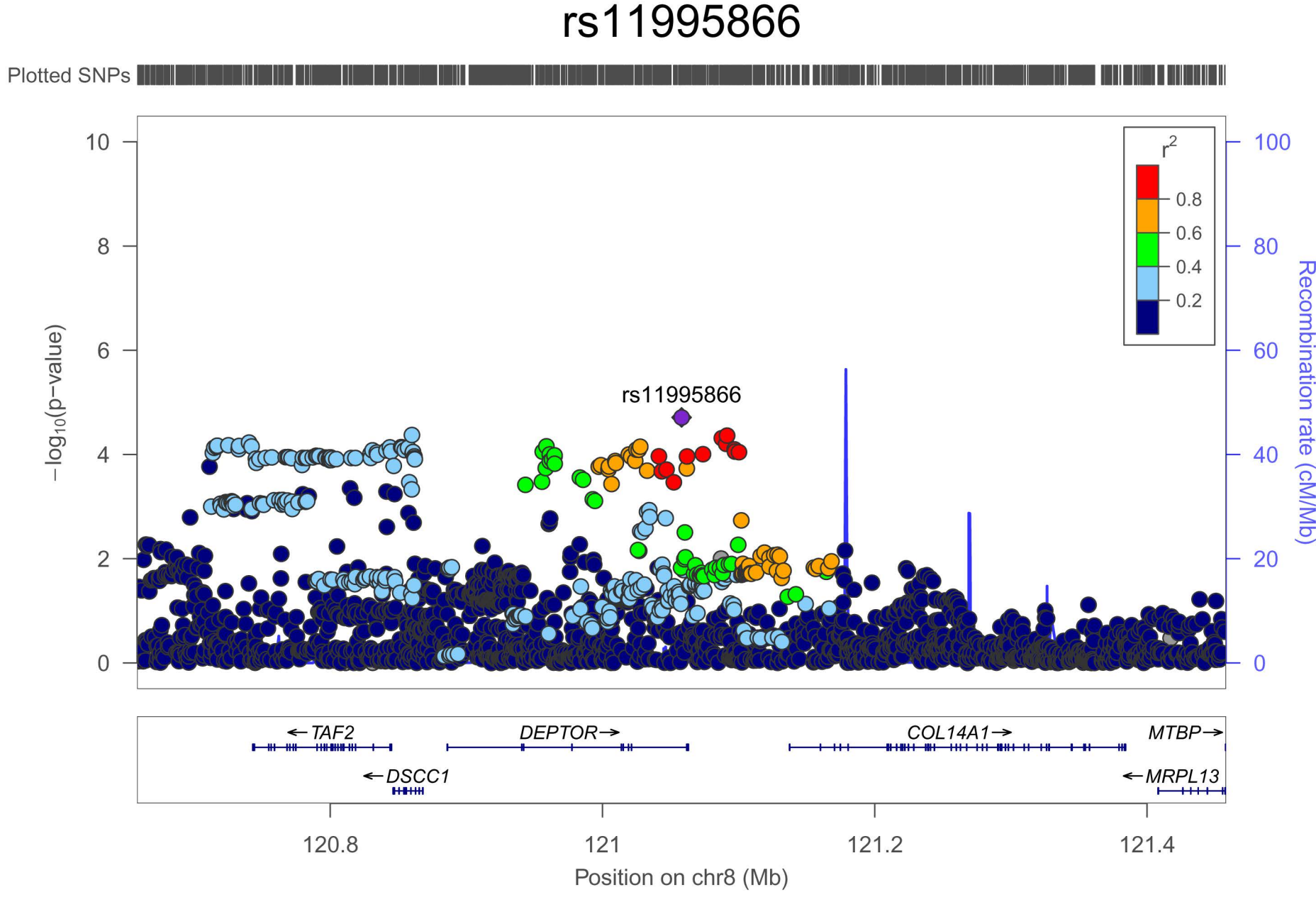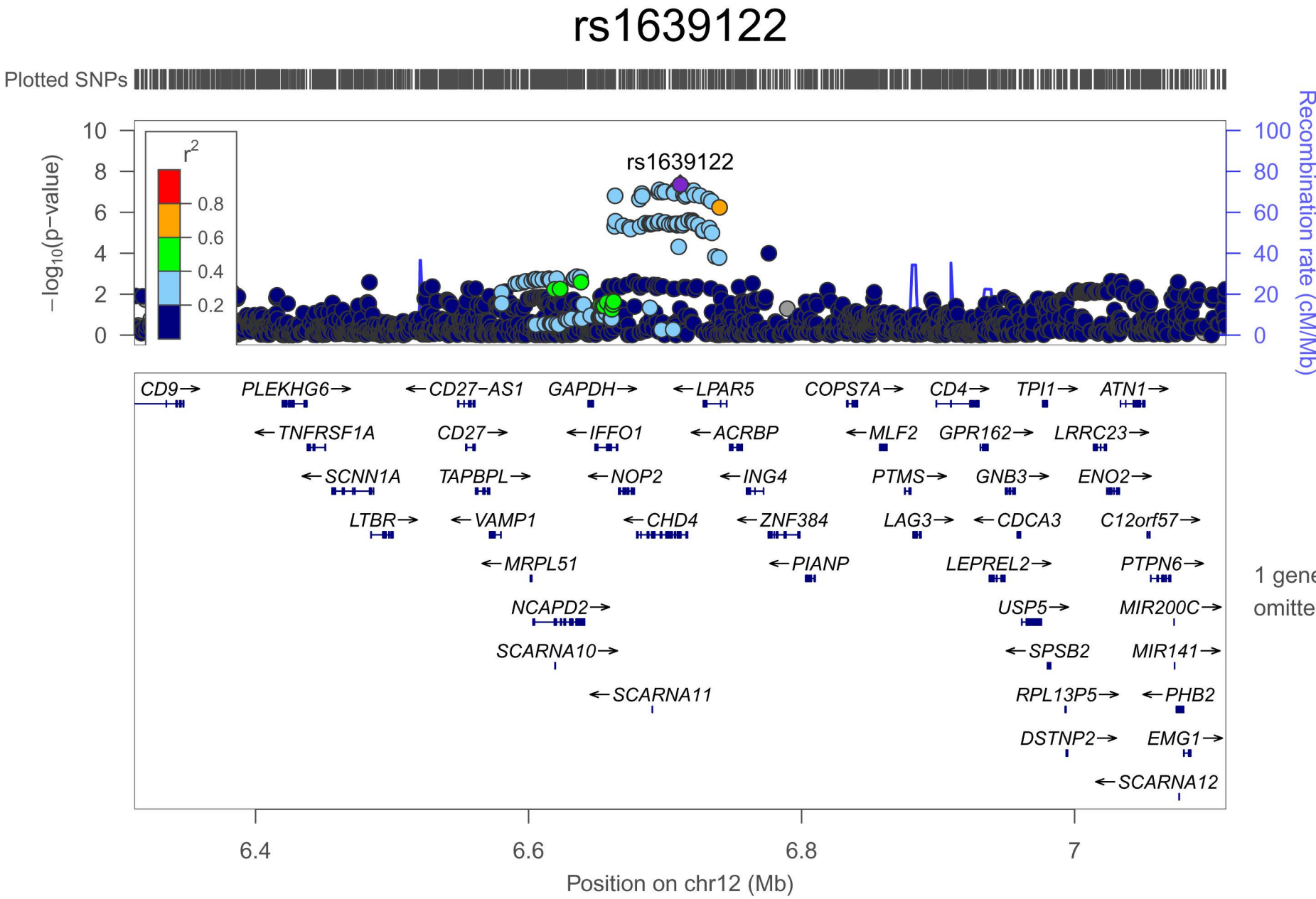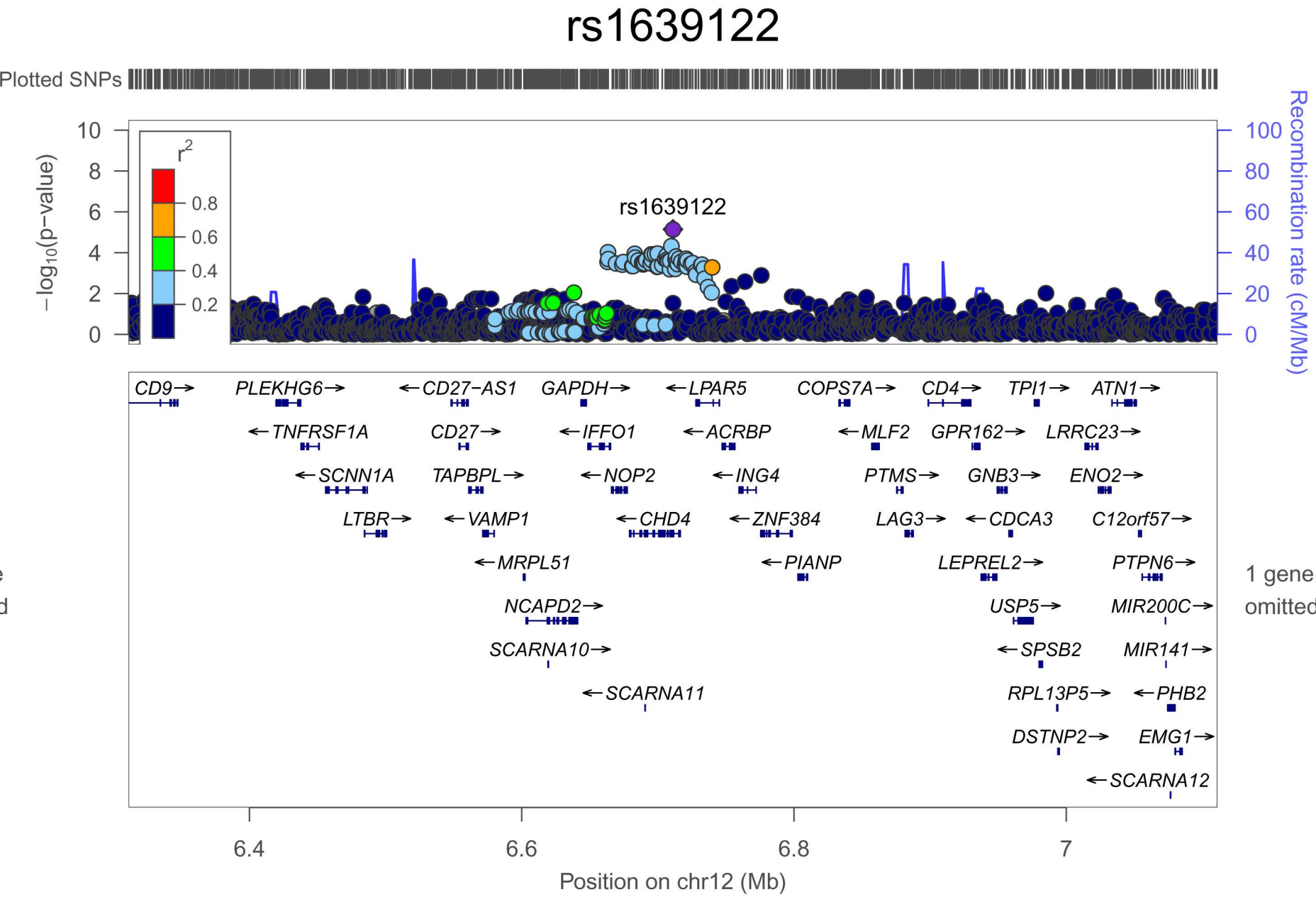

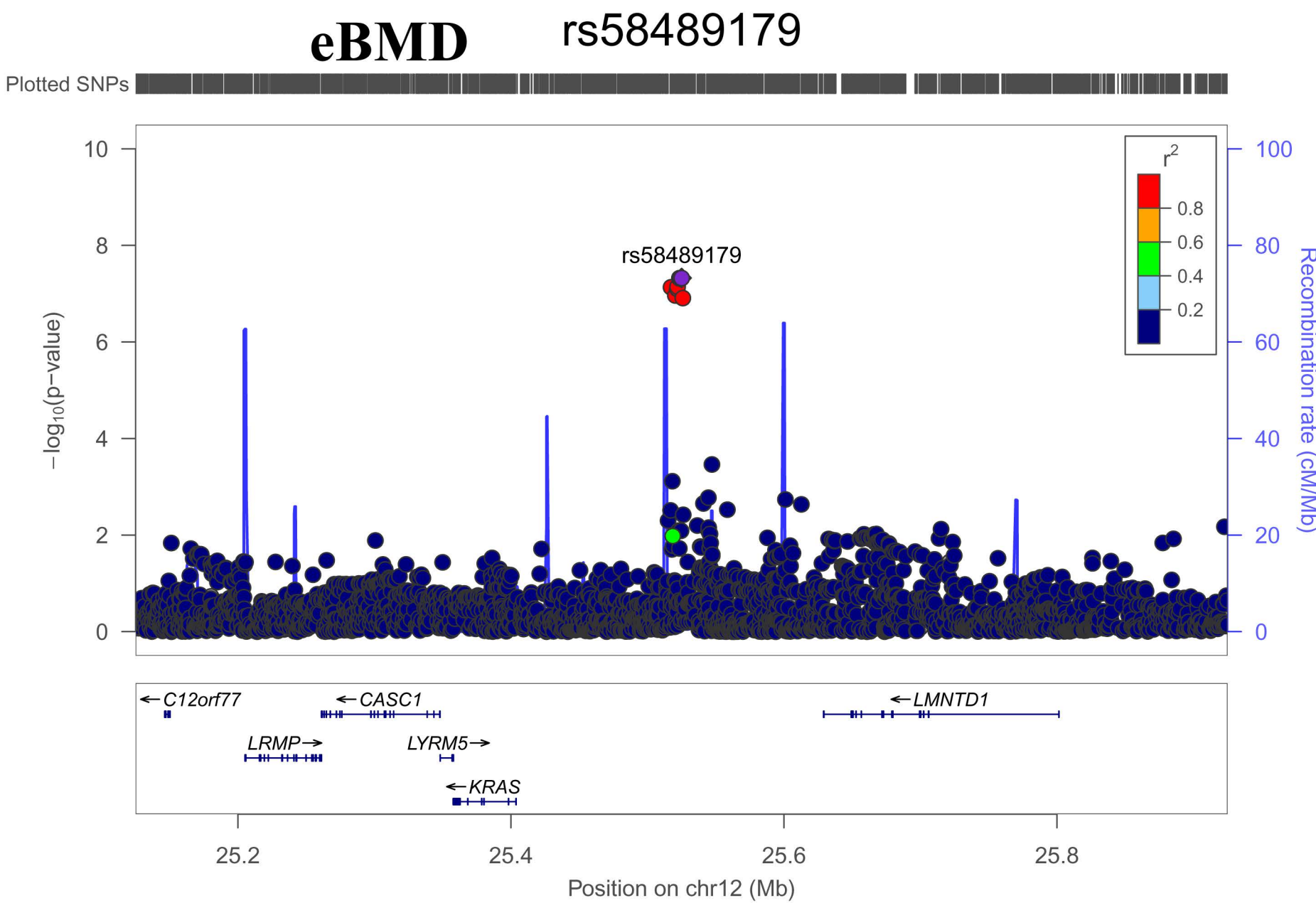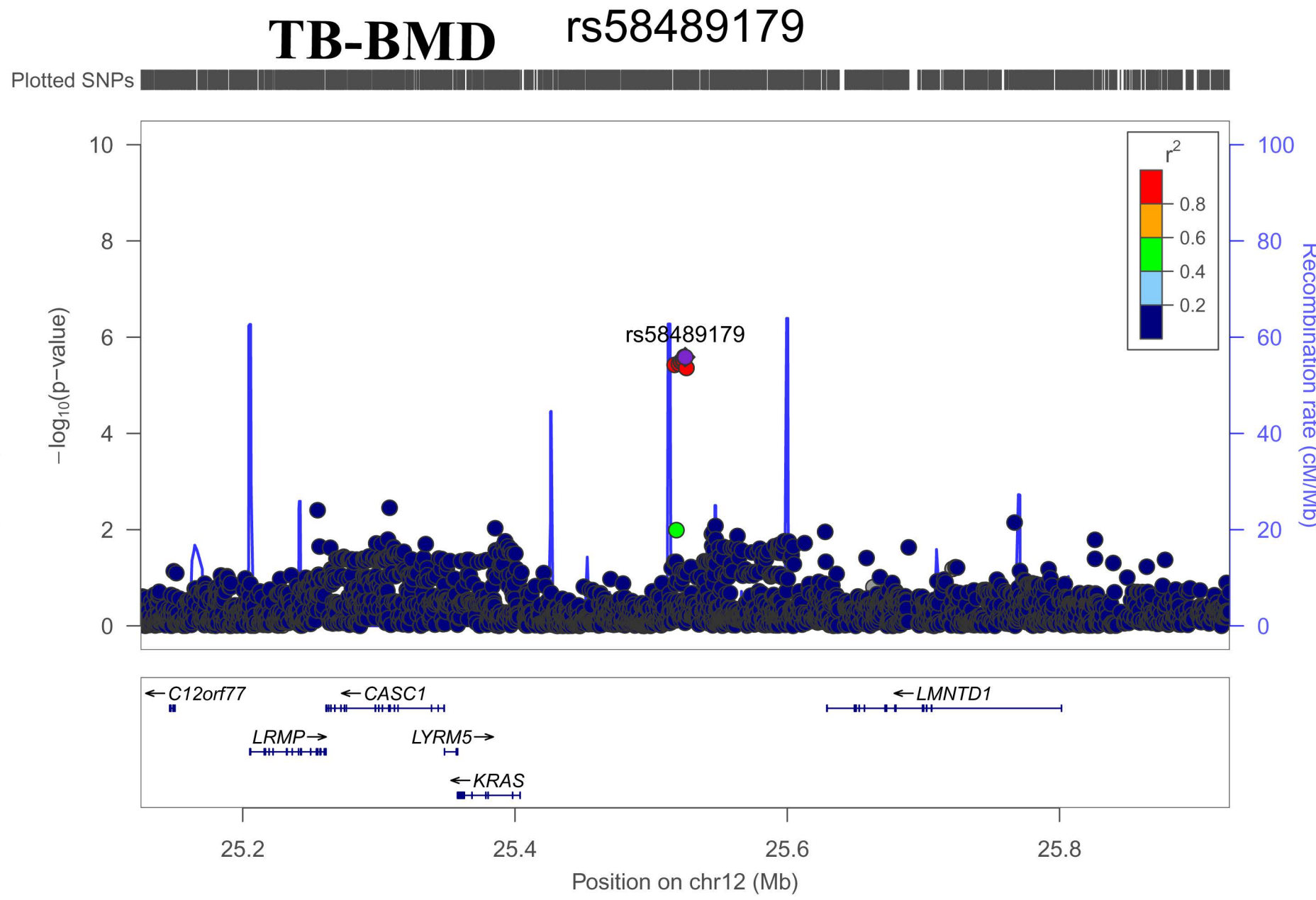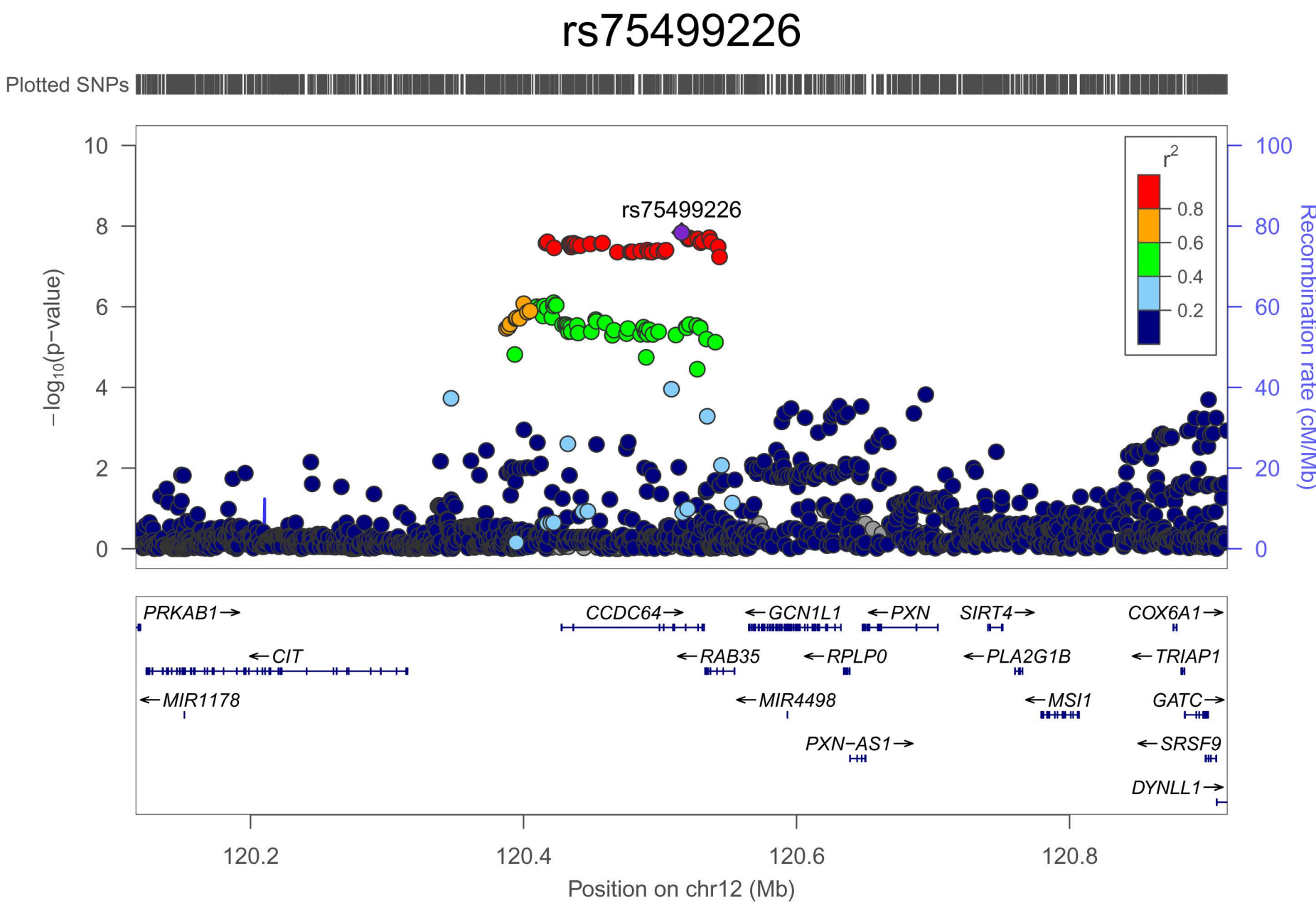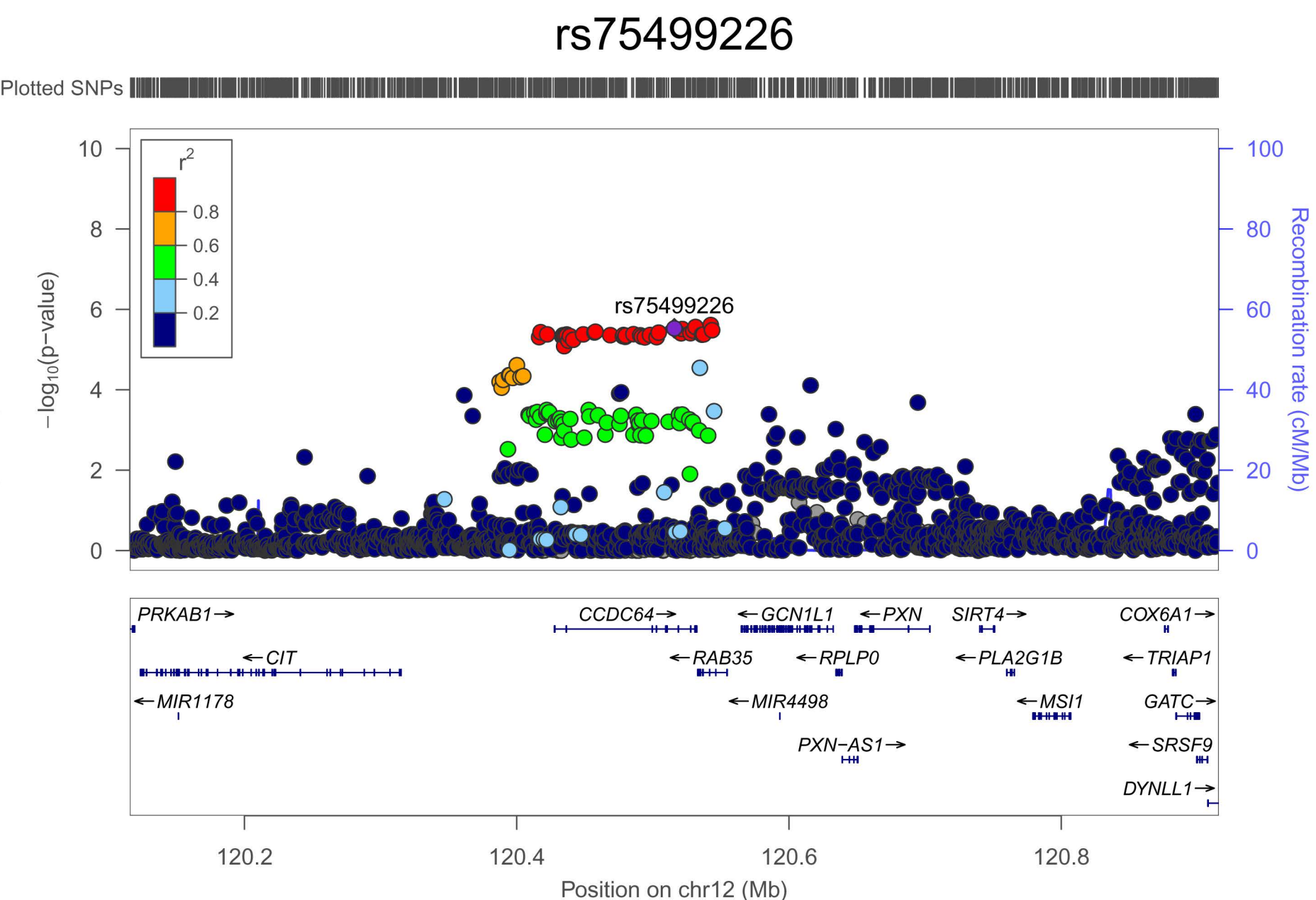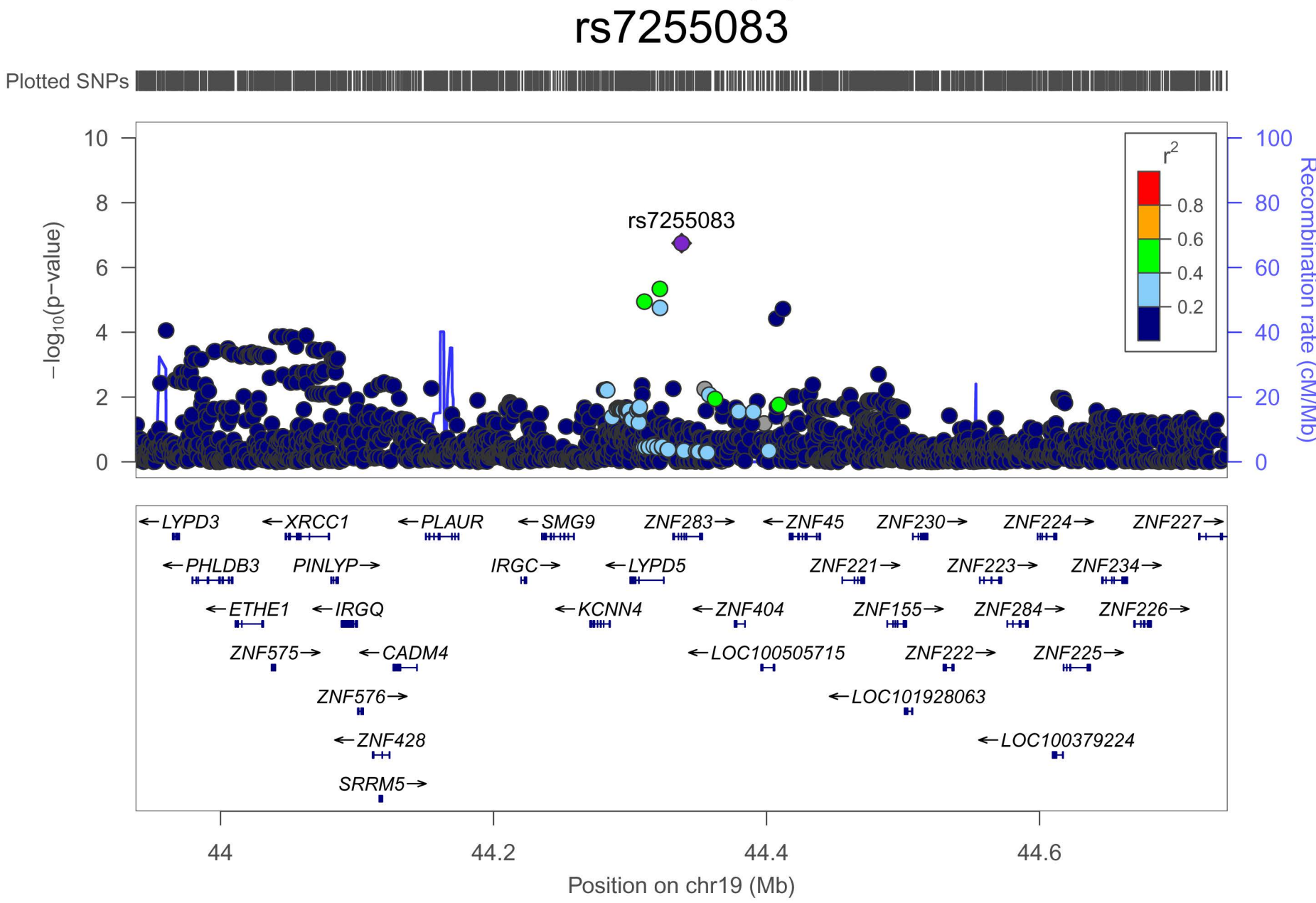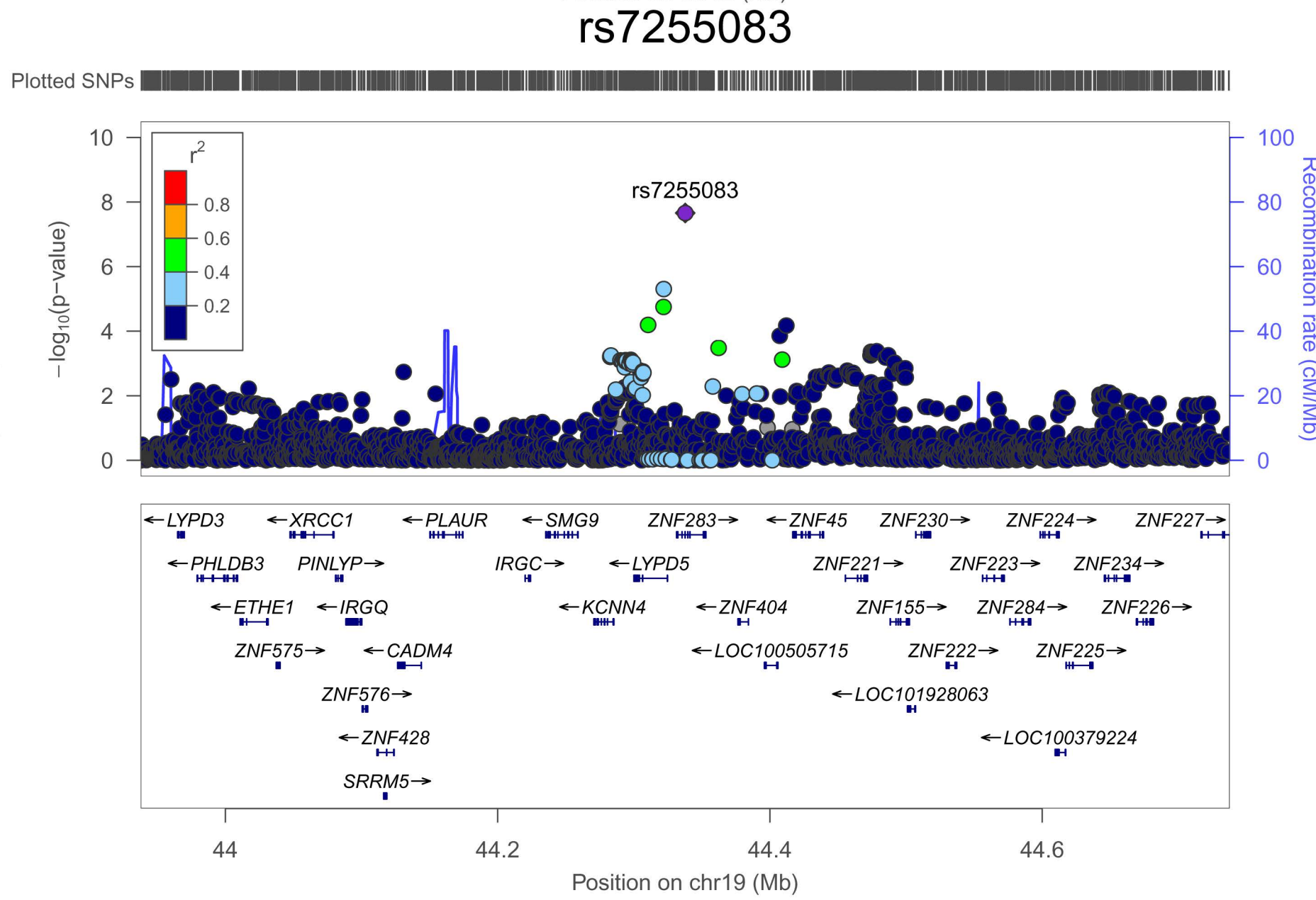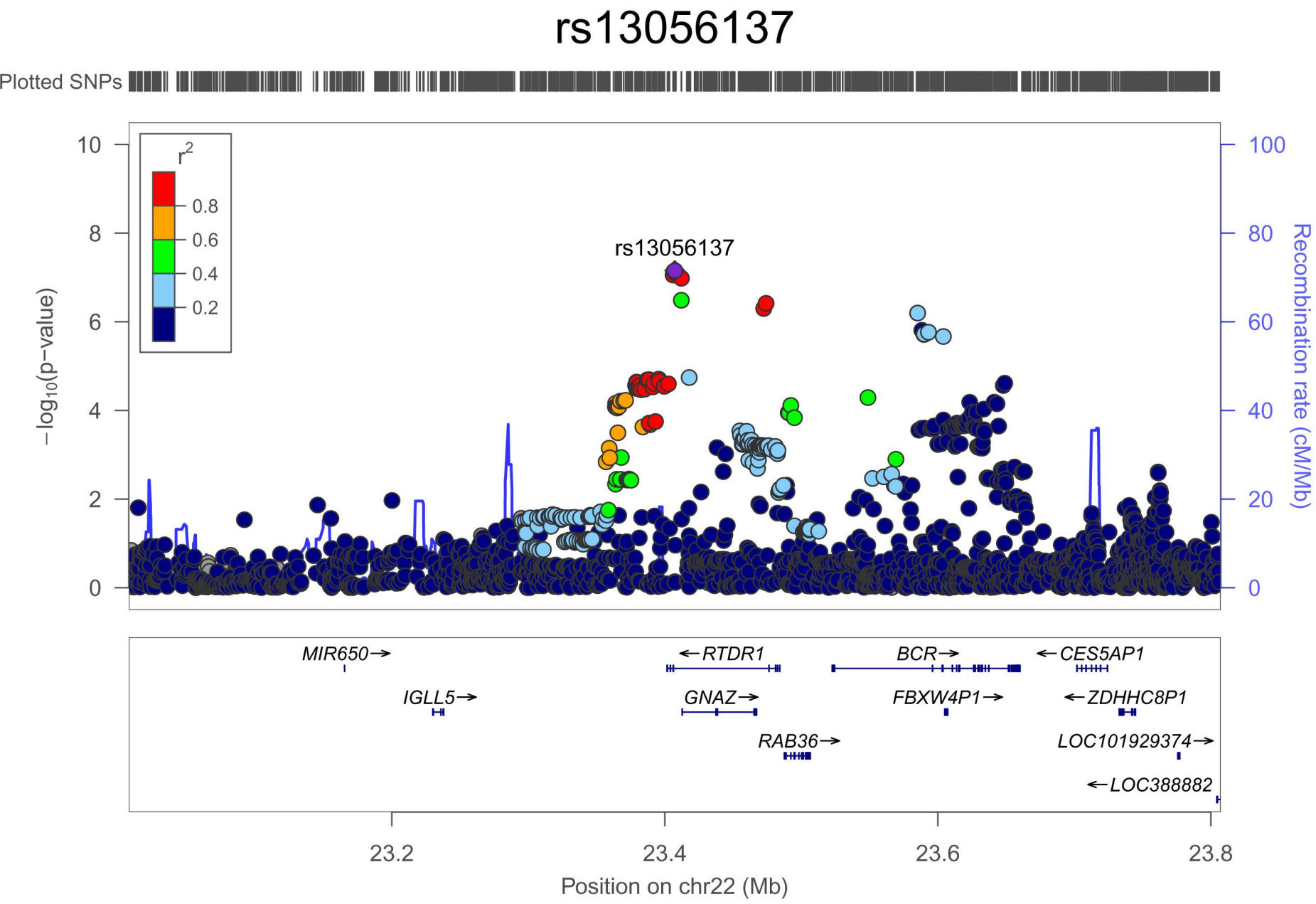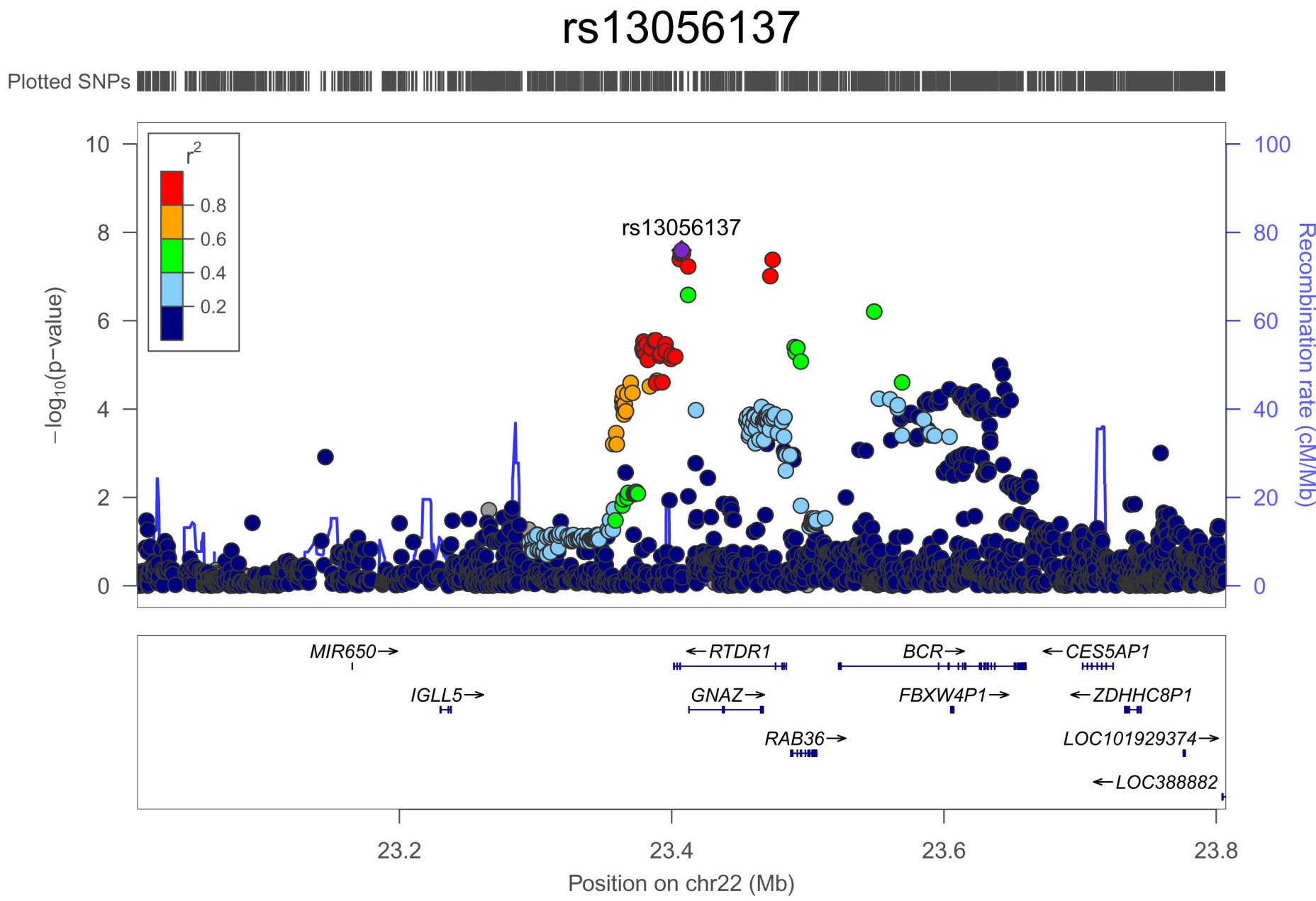

Supplement: Supplementary file 2 [file Data_Sheet_1.pdf]
